# Efficacy and safety of bevacizumab in Turkish patients with metastatic and recurrent cervical cancer

**DOI:** 10.4274/tjod.galenos.2020.90699

**Published:** 2020-07-29

**Authors:** Özlem Ercelep, Deniz Tataroğlu, Melike Özçelik, Heves Sürmeli, Mustafa Değirmenci, Mevlüde İnanç, Mehmet Aliustaoğlu, Mahmut Gümüş

**Affiliations:** 1 Kartal Dr. Lütfi Kırdar Training and Research Hospital, Clinic of Medical Oncology, İstanbul, Turkey; 2 University of Health Sciences Turkey, Tepecik Training and Research Hospital, Clinic of Medical Oncology, İzmir, Turkey; 3 Kayseri Training and Research Hospital, Clinic of Medical Oncology, Kayseri, Turkey; 4 İstanbul Medeniyet Universtiy, Göztepe Training and Research Hospital, Clinic of Medical Oncology, İstanbul, Turkey

**Keywords:** Cervical cancer, bevacizumab, metastatic

## Abstract

**Objective:**

To evaluate the efficacy of bevacizumab a monoclonal, antivascular endothelial growth factor antibody in combination with cytotoxic chemotherapy in Turkish patients with recurrent and metastatic cervical cancer.

**Materials and Methods:**

Data of 64 patients with metastatic or recurrent cervical cancer, receiving bevacizumab with first-line cisplatin or carboplatin and paclitaxel chemotherapy between 2013 and 2017 were retrospectively evaluated.

**Results:**

The mean age of the patients was 49 years (range, 28-68), the median follow-up time was 12 months (range, 2-53), the median progression-free survival (PFS) was eight months, and the median overall survival (OS) was 23 months. All 64 patients received a median of 6 (range, 1-12) bevacizumab and 6 (range, 2-12) chemotherapy cycles. The chemotherapy regimens used with bevacizumab were cisplatin and paclitaxel in 31 (48%) and carboplatin and paclitaxel in 33 (52%) patients. The survival in patients treated with bevacizumab and cisplatin plus paclitaxel was better-particularly in patients with no previous cisplatin-based radiosensitizer therapy-than those treated with carboplatin, paclitaxel, and bevacizumab (p=0.023). The bevacizumab dose was 7.5 mg/kg in 30 patients (47%) and 15 mg/kg in 34 patients (53%) every 21 days. No significant difference was reported in the OS and the PFS between the two groups. While the most common all-grades adverse events were nausea, neutropenia, anemia, and peripheral sensory neuropathy, the most common grade ≥3 adverse events were neutropenia, anemia, and peripheral sensory neuropathy.

**Conclusion:**

Adding bevacizumab to platinum and paclitaxel chemotherapy in a case of metastatic or recurrent cervical cancer is an effective and tolerable treatment for Turkish patients.

**PRECIS:** Adding bevacizumab to chemotherapy in advanced stage cervical cancer is an effective and tolerable treatment for Turkish patients.

## Introduction

Cervical cancer is the third most commonly diagnosed gynecologic malignancy and the third most common cause of death among all gynecologic cancers in the United States^(1)^. In 2012, cervical cancer accounted for an estimated 528,000 new cancer cases and for 266,000 deaths worldwide^(2)^. However, it can be prevented and usually cured if detected early^(3)^. The therapeutic paradigms in the primary management of cervical cancer are well-established, that is, early lesions are usually treated surgically and locally advanced lesions are managed with concurrent cisplatin chemotherapy and pelvic radiation^(4,5)^. When women with metastatic disease (stage IV B) are treated with systemic cisplatin combination regimens, the median survival is only 9-10 months^(4,6,7)^.

Vascular endothelial growth factor (VEGF) signaling is an important target for cancer therapy because of its role in tumor angiogenesis and its potential role in tumor cell survival. Bevacizumab acts as a monoclonal antibody against VEGF^(8,9)^ and this has been supported by a Phase 3 trial in which the survival of the women with metastatic cervical cancer increased when first-line chemotherapy was added^(10)^. The current retrospective study was conducted to assess the tolerability, safety, and activity of bevacizumab-containing therapy in Turkish patients with advanced cervical cancer.

## Materials and Methods

Data of 64 patients with radiologically confirmed stage IVB, persistent or recurrent cervical cancer diagnosis, and undergoing platinum (cisplatin or carboplatin) plus paclitaxel and bevacizumab treatment between 2013 and 2017 were retrospectively evaluated.

The patients were divided into two categories: those who had previously received chemoradiotherapy (including cisplatin) and those who had not. The patients were classified according to the bevacizumab dose and chemotherapy regimen in which bevacizumab was added to cisplatin or carboplatin, and the metastatic region of the patients was lymphatic or visceral.

Ethical approval was received from the Kartal Dr. Lütfi Kırdar Training and Research Hospital, Non-interventional Clinical Research Ethics Committee (approval number: 2016/514/88/18, date: 29.07.2016). No patient consent was required.

### Statistical Analysis

The overall survival (OS) and the progression-free survival (PFS) were calculated using the Kaplan-Meier method starting from the first day of the chemotherapy. Data were expressed as mean, standard deviation, median lowest value, median highest value, frequency, and rates. All p-values were 2-sided, and p=0.05 was considered as statistically significant. Data were analyzed using the SPSS software version 22.

## Results

**Figure 1:**
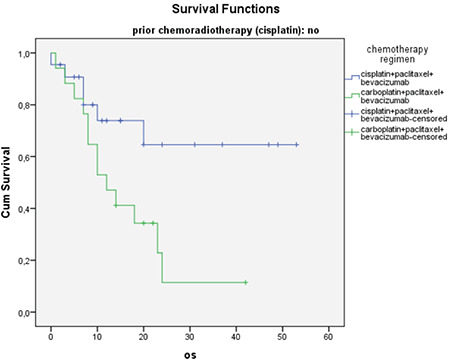
Overall survival according to chemotherapy regimen (prior chemoradiotherapy absent subgroup) OS: Overall survivol

**Figure 2:**
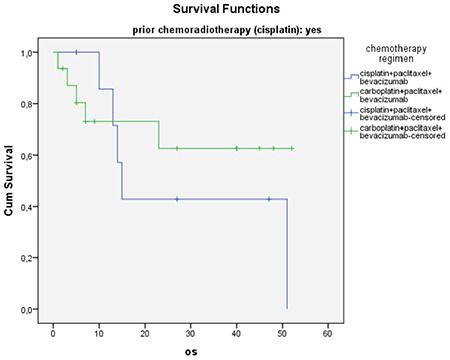
Overall survival according to chemotherapy regimen (prior chemoradiotherapy present subgroup) OS: Overall survival

The mean age of the 64 patients whose data were included in this study was 49 years (range, 28-68) (Table 1), and the median follow-up time was 12 months (range, 2-53). While the median PFS was 8 months, the median OS was 23 months (Table 2). The 64 treated patients received a median of six (range, 1-12) bevacizumab cycles and six (range, 2-12) chemotherapy cycles. Of those, 25 patients (39%) had previously received chemoradiotherapy with cisplatin. The survival with cisplatin, paclitaxel, and bevacizumab treatment was better in patients without a previous cisplatin-based radiosensitizer therapy than those treated with carboplatin, paclitaxel, and bevacizumab (p=0.02). There was no difference in the survival between the two regimens in patients who had previously received cisplatin (p=0.55) (Figures 1, 2).

The recommended bevacizumab dose was 7.5 mg/kg in 30 patients (47%) and 15 mg/kg in 34 patients (53%) every 21 days. No significant difference was observed in the OS and the PFS between the two groups. While 17 patients (26%) had metastases at the time of diagnosis and the median survival for them was 10 months, 47 patients (74%) had recurrent disease with the median survival of 24 months (p=0.14). The metastatic regions were visceral organs in 25 (39%) and lymphatic region in 39 patients (61%). The median survival was 10 months in patients with visceral and 51 months in those with lymphatic metastases (p=0.10).

While the most common all-grade adverse events were nausea, neutropenia, anemia, and peripheral sensory neuropathy, the most common grade ≥3 adverse events were neutropenia, anemia, and peripheral sensory neuropathy. Fistula and toxic death were not observed (Table 3).

## Discussion

This retrospective study was conducted with an aim to assess the tolerability, safety, and activity of bevacizumab-containing therapy in Turkish patients with advanced cervical cancer. Although, chemotherapy remains the standard treatment for patients with stage IVB, persistent or recurrent cervical cancer, no long-term disease control is achieved through this treatment^(11)^. Based on the results of the GOG204 and JCOG0505 studies, paclitaxel/cisplatin and paclitaxel/carboplatin are considered to be the recommended treatments for patients with recurrent cervical cancer^(12,13)^. VEGF plays a key role in the angiogenesis of the tumor, and although it is directly related to the extent of the disease, it is inversely related to the survival^(14)^. Bevacizumab is an antibody that recognizes and neutralizes all major isoforms of VEGF, inhibits endothelial cell proliferation, and inhibits receptor binding and vascular formation^(15)^.

In this study, the median PFS was reported as 8 months and the median OS as 23 months, and the overall response rate was 78%. About 39% of the patients had previously received a platinum-based radiosensitizer therapy. The survival with cisplatin, paclitaxel, and bevacizumab treatment was better in patients without a previous cisplatin-based radiosensitizer therapy than those who were treated with carboplatin plus paclitaxel and bevacizumab (p=0.023). No difference was observed in the survival between the two regimens in patients who had previously received cisplatin (p=0.556). Also, there was no significant difference in the OS and the PFS between patients with bevacizumab doses of 7.5 mg/kg (n=30) and patients with 15 mg/kg (n=34). The survival was 10 months in patients with systemic and 51 months in patients with lymphatic metastases. Although there was a numeric difference between the two groups, there was no statistical significance. This may be due to the insufficient number of patients (p=0.10). There was no toxic death due to treatment, no patient developed fistula, and grade ≥3 and higher side effects were in-line with the literature.

The efficacy of bevacizumab has been seen in a wide range of solid tumor types, including in case of advanced cervical cancer. Wright et al.^(16)^ in 2006 retrospectively evaluated 6 patients who had previously received median 3 systemic treatments. The authors reported that patients receiving bevacizumab with chemotherapy had a response rate of 67% and a median time of 4.3 months to tumor progression. Therefore, in this small cohort study, bevacizumab was well-tolerated.

In the first Phase 2 study conducted by Monk et al.^(17)^ in 2009, the efficacy of bevacizumab alone in patients receiving first- or second lines of systemic therapy for recurrent disease was investigated. The PFS in the study was reported as 3.4 months and the OS as 7.29 months. It was therefore shown to be an effective and well-tolerated treatment in the second- and third lines of therapy.

Later, in 2011, Schefter et al.^(18)^ conducted Phase 2 and 3 studies. In this study, 49 local advanced (stage IB-IIIB) patients were treated with chemoradiotherapy and bevacizumab 10 mg/kg every 2 weeks. Researchers concluded that the addition of bevacizumab in chemoradiotherapy is a feasible and safe treatment.

In Phase 3 trial, the investigators randomly assigned 452 patients to chemotherapy with or without bevacizumab at a dose of 15 mg/kg. Chemotherapy consisted of cisplatin plus paclitaxel or topotecan, and paclitaxel. Topotecan/paclitaxel was not superior to cisplatin/paclitaxel. Bevacizumab, as compared with chemotherapy alone, was associated with an increased incidence of grade 2 or higher hypertension, grade 3 or higher thromboembolic event, and grade 3 or higher gastrointestinal fistulas. The addition of bevacizumab to a combination chemotherapy in patients with recurrent, persistent, or metastatic cervical cancer was associated with an improvement of 3.7 months in median OS^(10)^.

Further, in 2016, Sugiyama et al.^(19)^ showed that cisplatin plus paclitaxel and bevacizumab treatment are effective, tolerable, and safe in Japanese patients with a small single-arm study (seven patients).

Although, owing to cytological screening and DNA testing for high-risk human papilloma virus types, the rate of cervical cancer has significantly reduced in developed countries, it, however, remains a major problem in undeveloped countries. If diagnosed at an early stage, patients can be cured by surgery, radiotherapy, and chemoradiotherapy treatments. However, in patients with recurrent metastatic disease or after platinum therapy, survival is poor with systemic treatments, and most cases cannot be cured. The newly targeted therapies and immunotherapy trials in advanced-stage patients continue to produce better survival outcomes and cure. Preventive vaccination and early detection of the disease are very important factors in survival. There is no study in the literature that compares carboplatin-paclitaxel and bevacizumab treatment with cisplatin-paclitaxel and bevacizumab treatment in patients with cervical cancer, and there is no study comparing the dose of bevacizumab 7.5 mg/kg with a dose of 15 mg/kg. In this retrospective study, we aimed to report our experience with such subjects and the efficacy and safety data of bevacizumab in our patient population.

### Study Limitations

Being a retrospective study, covering small number of patients and short follow-up time are the limitations of the study.

## Conclusion

The introduction of bevacizumab has been one of the most important recent advances for patients with advanced-stage cervical cancer. It is an effective and tolerable treatment for Turkish population with metastatic or recurrent cervical cancer.

## Figures and Tables

**Table 1:**
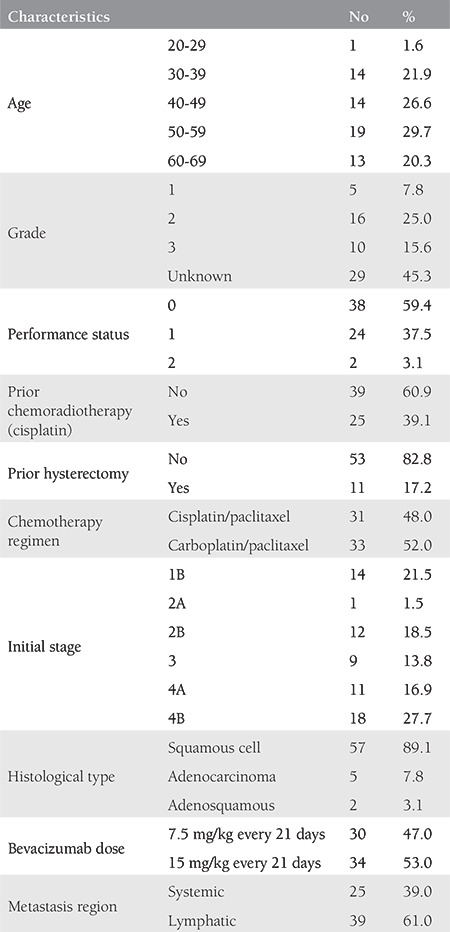
Clinicopathologic and treatment characteristics

**Table 2:**
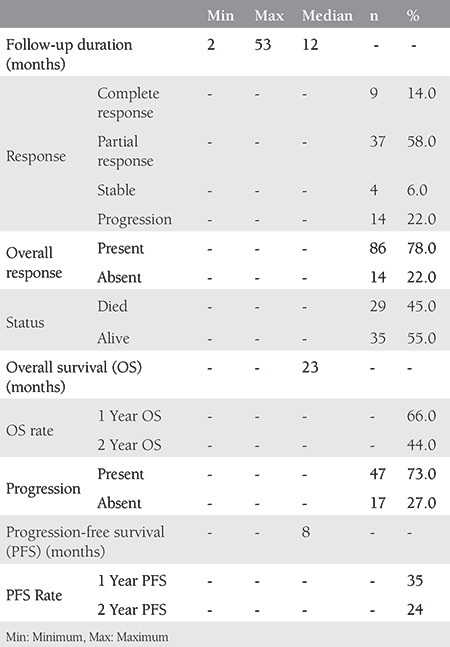
Follow-up and survival characteristics

**Table 3:**
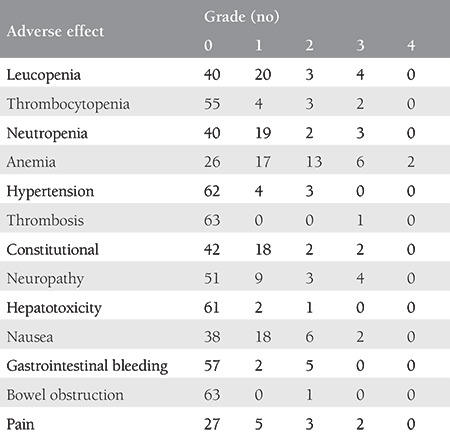
Adverse events

## References

[1] Siegel R, Ward E, Brawley O, Jemal A (2011). Cancer statistics, 2011: The impact of eliminating socioeconomic and racial disparities on premature cancer deaths. CA Cancer J Clin.

[2] Cervical cancer. Estimated incidence, mortality and prevalence worldwide in 2012. (Accessed on March 18, 2015)..

[3] Monk BJ, Herzog TJ (2007). The evolution of cost-effective screening and prevention of cervical carcinoma: Implications of the 2006 consensus guidelines and human papillomavirus vaccination. Am J Obstetric Gynecology.

[4] Monk BJ, Tewari KSL (2007). Chapter 3: Invasive cervical cancer, in DiSaia PJ, Creasman WT (eds): Clinical Gynecologic Oncology (ed 7). New York, NY, Mosby, Inc.

[5] Monk BJ, Tewari KS, Koh WJ (2007). Multimodality therapy for locally advanced cervical carcinoma: State of the art and future directions. J Clin Oncol.

[6] Tewari KS, Monk BJ (2005). Gynecologic oncology group trials of chemotherapy for metastatic and recurrent cervical cancer. Curr Oncol Rep.

[7] Long HJ III, Bundy BN, Grendys EC, Benda JA, McMeekin DS, Sorosky J, et al (2005). Randomized phase III trial of cisplatin with or without topotecan in carcinoma of the uterine cervix: A Gynecologic Oncology Group study. J Clin Oncol.

[8] Folkman J, D’Amore PA (1996). Blood vessel formation: What is its molecular basis?. Cell.

[9] Hlatky L, Hahnfeldt P, Tsionou C, Coleman CN (1996). Environmental controls and effects in angiogenesis. Br J Cancer.

[10] Tewari KS, Sill MW, Long HJ, Penson RT, Huang H, Ramondetta LM, et al (2014). Improved survival with bevacizumab in advanced cervical cancer. N Engl J Med.

[11] Greer BE, Koh WJ, Abu-Rustum NR, Apte SM, Campos SM, Chan J, et al (2010). Cervical cancer. J Natl Compr Canc Netw.

[12] Monk BJ, Sill MW, McMeekin DS, Cohn DE, Ramondetta LM, Boardman CH, et al (2009). Phase III trial of four cisplatin-containing doublet combinations in stage ıvb, recurrent, or persistent cervical carcinoma: A Gynecologic Oncology Group Study. J Clin Oncol.

[13] Saito I, Kitagawa R, Fukuda H, Shibata T, Katsumata N, Konishi I, et al (2010). A randomized, phase III trial of paclitaxel plus carboplatin (TC) versus paclitaxel plus cisplatin (TP) in stage IVb, persistent or recurrent cervical cancer: Japan Clinical Oncology Group study (JCOG0505). J Clin Oncol.

[14] Leung DW, Cachianes G, Kuang WJ, Goeddel DV, Ferrara N (1989). Vascular endothelial growth factor is a secreted angiogenic mitogen. Science.

[15] Ferrara N, Hillan KJ, Gerber HP, Novotny W (2004). Discovery and development of bevacizumab, an anti-VEGF antibody for treating cancer. Nat Rev Drug Discov.

[16] Wright JD, Viviano D, Powell MA, Gibb RK, Mutch DG, Grigsby PW, et al (2006). Bevacizumab combination therapy in heavily pretreated, recurrent cervical cancer. Gynecol Oncol.

[17] Monk BJ, Sill MW, Burger RA, Gray HJ, Buekers TE, Roman LD (2009). Phase II trial of bevacizumab in the treatment of persistent or recurrent squamous cell carcinoma of the cervix: a Gynecologic Oncology Group Study. J Clin Oncol.

[18] Schefter TE, Winter K, Kwon JS, Stuhr K, Balaraj K, Yaremko BP, et al (2012). A phase II study of bevacizumab in combination with definitive radiotherapy and cisplatin chemotherapy in untreated patients with locally advanced cervical carcinoma: preliminary results of RTOG 0417. Int J Radiat Oncol Biol Phys.

[19] Sugiyama T, Mizuno M, Aoki Y, Sakurai M, Nishikawa T, Ueda E, et al (2017). A single-arm study evaluating bevacizumab, cisplatin, and paclitaxel followed by single-agent bevacizumab in Japanese patients with advanced cervical cancer. Jpn J Clin Oncol.

